# How Effective Are Machine Learning and Doubly Robust Estimators in Incorporating High‐Dimensional Proxies to Reduce Residual Confounding?

**DOI:** 10.1002/pds.70155

**Published:** 2025-05-14

**Authors:** Mohammad Ehsanul Karim, Yang Lei

**Affiliations:** ^1^ School of Population and Public Health University of British Columbia Vancouver British Columbia Canada; ^2^ Centre for Advancing Health Outcomes University of British Columbia Vancouver British Columbia Canada; ^3^ Department of Statistics University of British Columbia Vancouver British Columbia Canada

**Keywords:** hdPS, high‐dimension, plasmode simulation, proxy adjustment, TMLE

## Abstract

**Background:**

Residual confounding presents a persistent challenge in observational studies, particularly in high‐dimensional settings. High‐dimensional proxy adjustment methods, such as the high‐dimensional propensity score (hdPS), are widely used to address confounding bias by incorporating proxies for unmeasured confounders. Extensions of hdPS have integrated machine learning, such as LASSO and super learner (SL), and doubly robust estimators, such as targeted maximum likelihood estimation (TMLE). However, the comparative performance of these methods, especially under different learner configurations and high‐dimensional proxies, remains unclear.

**Method:**

We conducted plasmode simulations to evaluate the performance of standard methods, SL, TMLE, and double cross‐fit TMLE (DC‐TMLE) under varying exposure and outcome prevalence scenarios. Learner libraries included: 1 learner (logistic regression), 3 learners (logistic regression, MARS, and LASSO), and 4 learners (adding XGBoost, a non‐Donsker learner). Metrics included bias, coverage, and variability.

**Results:**

Methods without proxies exhibited the highest bias and poorest coverage, highlighting the critical role of proxies in confounding adjustment. Standard methods incorporating high‐dimensional proxies showed robust performance, achieving low bias and near‐nominal coverage. TMLE and DC‐TMLE reduced bias but exhibited worse coverage compared to standard methods, particularly with larger learner libraries. Notably, DC‐TMLE, expected to address under‐coverage issues, failed to perform adequately in high‐dimensional settings with non‐Donsker learners, further emphasizing the instability introduced by complex libraries.

**Conclusion:**

Our findings underscore the utility of high‐dimensional proxies in standard methods and the importance of tailoring learner configurations in SL and TMLE to ensure reliable confounding adjustment in high‐dimensional contexts.

**Summary**

- Methods that incorporate high‐dimensional proxies significantly improve bias reduction and coverage compared to methods without proxies, emphasizing their importance in confounding adjustment.
- Super learner (SL) and targeted maximum likelihood estimation (TMLE) enhance the flexibility of confounding adjustment, but their performance varies depending on the choice of learners.
- While diverse learner libraries offer flexibility, overly complex configurations—particularly those including non‐Donsker learners such as XGBoost—can lead to increased bias and poor coverage in high‐dimensional settings.
- Traditional high‐dimensional propensity score (hdPS) methods or machine learning alternatives such as LASSO, when incorporating high‐dimensional proxies, have the potential to perform comparably or better than more complex approaches, offering a stable and interpretable alternative.
- The effectiveness of confounding adjustment methods depends not only on model flexibility, but also on careful variable selection and stability considerations, particularly in high‐dimensional data settings common in pharmacoepidemiology.

**Abbreviations**
CI: confidence interval
C‐TMLE: collaborative targeted maximum likelihood estimation
CVTMLE: cross‐validated targeted maximum likelihood estimation
DC‐TMLE: double cross‐fit targeted maximum likelihood estimation
hdPS: high‐dimensional propensity score
IPW: inverse probability weighting
LASSO: least absolute shrinkage and selection operator
MARS: multivariate adaptive regression splines
ML: machine learning
MSE: mean squared error
PS: propensity score
RD: risk difference
RMSE: root mean squared error
SE: standard error
SL: super learner
TMLE: targeted maximum likelihood estimation
XGBoost: extreme gradient boosting

## 1 Background

### 1.1 High‐Dimensional Propensity Score

Residual confounding is pervasive in analyses of health administrative databases, where unmeasured or imperfectly measured confounders can bias effect estimates. The high‐dimensional propensity score (hdPS) method is widely used in pharmacoepidemiology and health outcomes research to leverage large administrative databases capturing diverse healthcare interactions (e.g., diagnostic codes, prescriptions, procedures) [1]. Unlike traditional propensity score approaches that rely exclusively on pre‐specified covariates [2], hdPS systematically identifies and includes proxy confounders from high‐dimensional data [3]. Known confounders (e.g., those in causal diagrams) are always retained a priori, ensuring that critical variables are never subjected to data‐driven selection. By integrating prior knowledge with automated proxy selection, hdPS improves control of both measured and unmeasured confounding, capitalizing on the richness of large healthcare datasets while maintaining interpretability.

### 1.2 Extensions and Gaps

The original hdPS algorithm uses Bross's formula to rank potential proxies according to their marginal associations with treatment and outcome [4]. However, high‐dimensional and correlated administrative data can lead to overfitting and multicollinearity. To address these issues, multi‐variate and machine learning alternatives have been proposed [5]. Examples include regularized regression (e.g., LASSO, elastic net) [6, 7], tree‐based methods [7, 8] and ensemble methods (e.g., super learner) that incorporate flexible modeling strategies [9, 10].

Some studies have used targeted maximum likelihood estimation (TMLE), which combines flexible machine learning for both treatment and outcome models. However, logistic regression is still commonly chosen as the sole learner within TMLE applications for computational reasons [11, 12]. This means the full potential of TMLE with a diverse super learner remains a relatively unexplored area in the hdPS context. While double‐robust methods (e.g., TMLE) have outperformed singly robust approaches in low‐dimensional settings with investigator‐specified covariates [13, 14], it remains unclear whether these findings extend to high‐dimensional contexts, especially when the number of potential proxies is large (possibly including noise variables) and model specification is complex. Moreover, the performance of TMLE can depend on the choice of candidate learners in the super learner [15], and additional sample‐splitting (e.g., double cross‐fitting) may be required to ensure robust estimation [16, 17]. However, the computational burden and practical positivity challenges of double cross‐fitting approaches can be substantial, particularly in large datasets. This may explain the lack of implementation of these newly proposed double cross‐fitting methods in the hdPS literature.

### 1.3 Aim

Despite numerous proposed enhancements to hdPS‐related methods, critical gaps remain in understanding their comparative performance when integrated with machine learning and doubly robust estimators. Specifically, evaluating how these approaches perform under varying data complexities, exposure and outcome prevalence, and different candidate learner configurations is essential for guiding practitioners in designing robust analytical protocols. This study aims to address these gaps by systematically assessing these methods under realistic conditions using plasmode simulations. To further contextualize our findings, we will apply these methods to real‐world data from the National Health and Nutrition Examination Survey (NHANES) [18] (2013–2018), providing empirical insights into their practical applicability.

## 2 Methods

### 2.1 Motivating Real‐Wold Example

Obesity is a well‐established risk factor for type 2 diabetes, with excess body fat contributing to insulin resistance and impaired blood sugar regulation [19]. To investigate this relationship, we will use data from NHANES. The dataset includes 7585 individuals [1], of whom 48.8% were categorized as obese (binary exposure) and 23.7% had diabetes (binary outcome).

The dataset includes 14 demographic, behavioral, and health history‐related variables (mostly categorical) and 11 laboratory‐measured variables (mostly continuous) as measured confounders. However, residual confounding may still arise from unmeasured comorbidities or other health conditions not captured in the survey. To address this limitation, we leverage the NHANES Prescription Medications component, which provides detailed data on prescription medications taken by participants prior to the survey. This information can serve as a crude proxy for comorbidity burden, helping to account for unmeasured health conditions [20, 21, 22].

In this context, methods under the hdPS framework have the potential to reduce residual confounding by systematically incorporating proxy information derived from prescription medication data. By integrating these proxies into the analysis, we aim to improve our estimates and better isolate the causal effect of obesity on diabetes.

### 2.2 Methods Under Consideration

We evaluated the association estimates using the hdPS method [3] and its alternatives, as summarized in Table 1 (see Supporting Information: Appendix §A for further details). Several of these approaches, such as LASSO [6, 7], super learner [9], and TMLE [11, 12], have been previously proposed and implemented in the literature. Standard methods, including hdPS, LASSO, and a hybrid hdPS‐LASSO approach, were implemented similarly to their original implementations in the literature [6, 7], with the exception that we used inverse probability weighting (IPW) instead of including propensity scores as covariates in the outcome regression.

**TABLE 1:** High‐dimensional propensity score and its machine learning and double robust alternatives.

Group	Method name	Description
Double cross‐fit TMLE	DC.TMLE	A double cross‐fit version of targeted maximum likelihood estimation (TMLE). Super learners were used to estimate propensity scores and outcomes, incorporating both investigator‐specified covariates and all proxies to estimate the treatment effect.
TMLE methods	hdPS.TMLE	High‐dimensional propensity score (hdPS) was used to select proxies, and TMLE was applied to estimate the treatment effect. Super learners were used for propensity score and outcome estimation, incorporating investigator‐specified covariates and hdPS‐selected proxies as input variables.
	LASSO.TMLE	Similar to hdPS.TMLE, but LASSO was used for proxy selection instead of hdPS.
	hdPS.LASSO.TMLE	Similar to hdPS.TMLE, but proxy selection was performed using a hybrid of hdPS and LASSO.
	TMLE.ks	Similar to hdPS.TMLE, but no proxy selection was conducted (all proxies were included in the model).
Super learner methods	hdPS.SL	Super learner approach using hdPS‐generated proxies as input, along with investigator‐specified covariates, to estimate the propensity score model. A variety of outcome models were used.
	LASSO.SL	Similar to hdPS.SL, but proxy selection was conducted using LASSO.
	hdPS.LASSO.SL	Similar to hdPS.SL, but proxy selection was conducted using a hybrid of hdPS and LASSO.
	SL.ks	Similar to hdPS.SL, but no proxy selection was conducted (all proxies were utilized).
Standard methods with proxies	PS.ks	Propensity score estimation using a “kitchen sink” approach, where all proxies were included. A variety of outcome models were used.
	hdPS	Traditional high‐dimensional propensity score approach, using the Bross formula to select proxies (100 proxy variables with the highest bias rankings). A variety of outcome models were used.
	LASSO	LASSO outcome regression was used for proxy selection (proxy variables with non‐zero coefficients). A variety of outcome models were used.
	hdPS.LASSO	Proxy selection was performed using a combination of hdPS and LASSO. A variety of outcome models were used.
Standard methods without proxies	TMLE.u	TMLE without using any proxies; only investigator‐specified covariates were included. A variety of outcome models were used.
	SL.u	Super learner without incorporating any proxies; only investigator‐specified covariates were included. A variety of outcome models were used.
	PS.u	Propensity score estimation using only investigator‐specified covariates, without incorporating proxies. A variety of outcome models were used.

*Note:* We employed three super learner libraries: a 1‐model library (logistic regression), a 3‐model library (logistic regression, LASSO, and MARS), and a 4‐model library (logistic regression, LASSO, MARS, and XGBoost).

Abbreviations: hdPS: high‐dimensional propensity score; ks: “kitchen sink” (all proxies included); LASSO: least absolute shrinkage and selection operator; PS: propensity score; SL: super learner; TMLE: targeted maximum likelihood estimation; u: unadjusted for high‐dimensional proxies.

For the non‐TMLE methods, we evaluated performance using different adjustment sets in the weighted outcome model to understand the impact of various levels of adjustment on bias and coverage. Five distinct adjustment strategies were considered: no adjustment, imbalanced measured confounders, all measured confounders, imbalanced measured confounders with proxies, and all measured confounders with proxies. The “no adjustment” scenario served as a baseline, with no confounders or proxies included, allowing us to observe the effects of residual confounding. For these analyses, we explored both stabilized and unstabilized weights to assess their potential impact on performance metrics.

However, implementations involving super learner and TMLE varied significantly across studies (because candidate learners were different), making direct comparisons challenging. For instance, some applications of super learner utilized 23 candidate learners [9], while others used only logistic regression for propensity score estimation [11, 12]. Additionally, methods such as double cross‐fitted TMLE have been implemented in low‐dimensional settings [16, 17] but remain largely unexplored in the hdPS context.

In our study, we employed three distinct versions of super learner for each analysis:

1. A 1‐model library consisting of logistic regression,
2. A 3‐model library including logistic regression, LASSO, and multivariate adaptive regression splines (MARS) and
3. A 4‐model library comprising logistic regression, LASSO, MARS, and extreme gradient boosting (XG‐Boost). XGBoost, a non‐smooth learner that does not belong to the Donsker class, was included only in the 4‐model library.

### 2.3 Plasmode Simulation

To rigorously evaluate the performance of the methods under consideration, we employed a plasmode simulation framework, which is particularly well‐suited for replicating real‐world data structures and complexities [23]. This approach was inspired by the analytic dataset derived from NHANES and involved resampling observed covariates and exposure information (i.e., obesity) without modification. By preserving key aspects of an actual epidemiological study, this simulation framework offers a significant advantage over traditional Monte Carlo simulations, which often rely on hypothetical assumptions.

#### 2.3.1 Simulation Scenarios

Our plasmode simulation was conducted over 500 iterations. For the base simulation scenario, we set the prevalence of exposure (obesity) and the event rate (diabetes) at 30%, with a true odds ratio (OR) of 1, corresponding to a risk difference (RD) of 0. Each simulated dataset included 3000 participants. Additional simulation scenarios, reflecting varying exposure and outcome prevalences, are summarized in Table 2.

**TABLE 2:** Overview of plasmode simulation scenarios reflecting varying exposure and outcome prevalences based on National Health and Nutrition Examination Survey (NHANES) data cycles (2013–2018).

Plasmode simulation scenario	Exposure prevalence	Outcome prevalence	True odds ratio	Sample size
(i) Frequent exposure and outcome (base)	30%	30%	1	3000
(ii) Rare exposure and frequent outcome	5%	30%	1	3000
(iii) Frequent exposure and rare outcome	30%	5%	1	3000

#### 2.3.2 Data‐Generating Mechanism

The primary goal of this plasmode simulation study was to evaluate various variable selection methods under realistic conditions. To achieve this, we formulated the outcome data based on a specific model specification incorporating both exposure and covariates, including investigator‐specified and proxy variables. The model specification consisted of three key components (see Supporting Information: Appendix §B for further details).

First, we retained the original investigator‐specified covariates, which included demographic, behavioral, and health history/access variables. These covariates were binary or categorical, reflecting how real‐world studies typically operate. They were not included in the pool of proxy variables or subjected to variable selection or ranking processes. Instead, they were always included in the analysis as a separate set of variables based on prior knowledge and causal reasoning, ensuring that known confounders were appropriately accounted for.

Second, to simulate the uncertainty often faced by analysts in real‐world studies, we transformed continuous laboratory variables using complex functions, such as logarithmic, exponential, square root, polynomial transformations, and interactions. This reflects the challenges analysts face in correctly specifying models when dealing with continuous data.

Third, we addressed the issue of unmeasured confounding by incorporating proxy variables [24]. Real‐world studies often contend with unmeasured confounding, which researchers attempt to mitigate by adding proxy variables. However, when a large number of proxies are added, some may act as noise variables, contributing little or nothing to the analysis. To simulate this, we selected binary proxy covariates that were highly associated with the outcome. Proxy variables were defined based on their association with the outcome because unmeasured confounders must influence the outcome to induce confounding. By prioritizing variables with strong outcome associations, we aimed to ensure that the proxies reflect at least part of the confounding structure. While not explicitly enforced, many of the selected proxy variables are expected to have some degree of association with the exposure.

Of the 142 proxy covariates, 94 met this criterion and were summed to calculate a simple comorbidity burden measure [25]. This score represents an individual's overall comorbidity burden, reflecting the cumulative presence of conditions. The remaining 48 covariates were excluded from this calculation and considered noise. This comorbidity burden measure (one variable) was then incorporated into our model specification for generating the outcome data.

#### 2.3.3 Performance Measures

From this simulation, we derived several performance metrics to evaluate the effectiveness of the methods under consideration through RD measures [26]. These metrics include bias, average model‐based standard error (SE) (the average of estimated SEs obtained from a model over repeated samples), empirical SE (the standard deviation of estimated treatment effects across repeated samples), mean squared error (MSE), coverage probability of 95% confidence intervals, bias‐corrected coverage, and Zip plot [27, 28].

All code used in this simulation study is publicly available at https://github.com/ehsanx/hdps‐tmle‐sim. The repository includes the complete simulation pipeline, analytic functions, and documentation to support replication of the analyses. Additionally, all simulation results can be interactively reviewed through a Shiny web application at https://ehsank.shinyapps.io/hdPS‐TMLE/.

## 3 Results

### 3.1 Motivating Example

**FIGURE 1:**
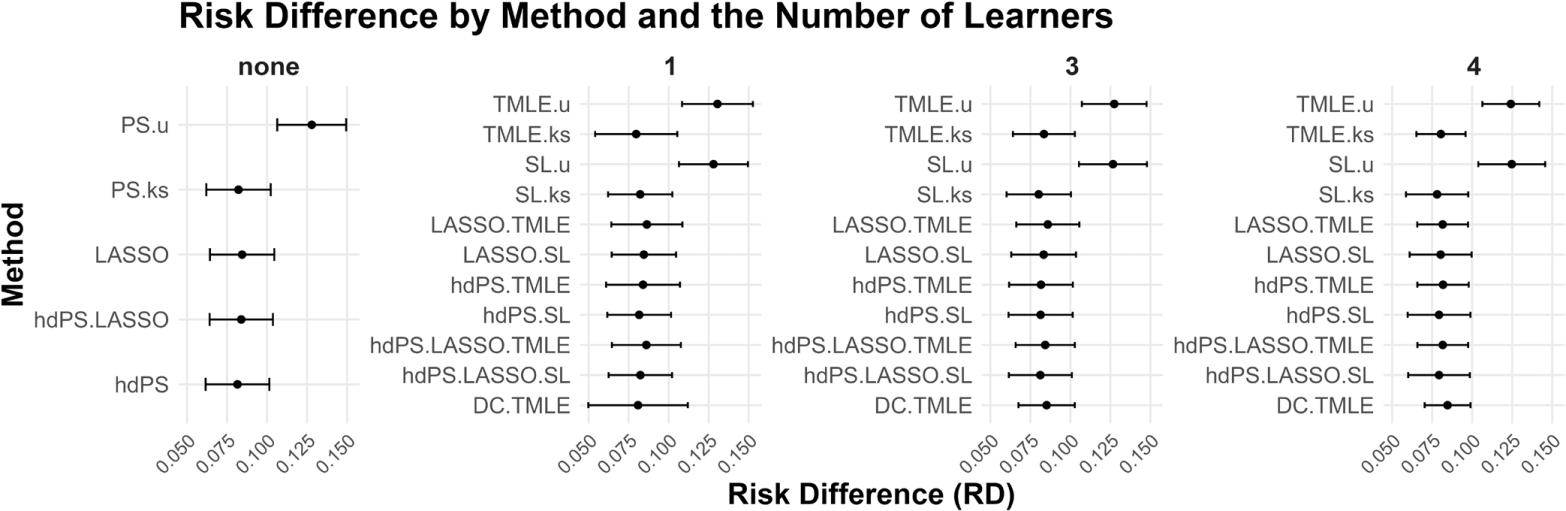
The association between obesity and diabetes in the National Health and Nutrition Examination Survey (NHANES) data (2013–2018). We employed three super learner libraries: A 1‐model library (logistic regression), a 3‐model library (logistic regression, LASSO, and MARS), and a 4‐model library (logistic regression, LASSO, MARS, and XGBoost).

The results are shown in Figure 1. Note that, other than the methods that ignored the proxies completely (with a *u* at the end), the rest of the methods provided very similar point estimates.

### 3.2 Plasmode Simulation Results

**FIGURE 2:**
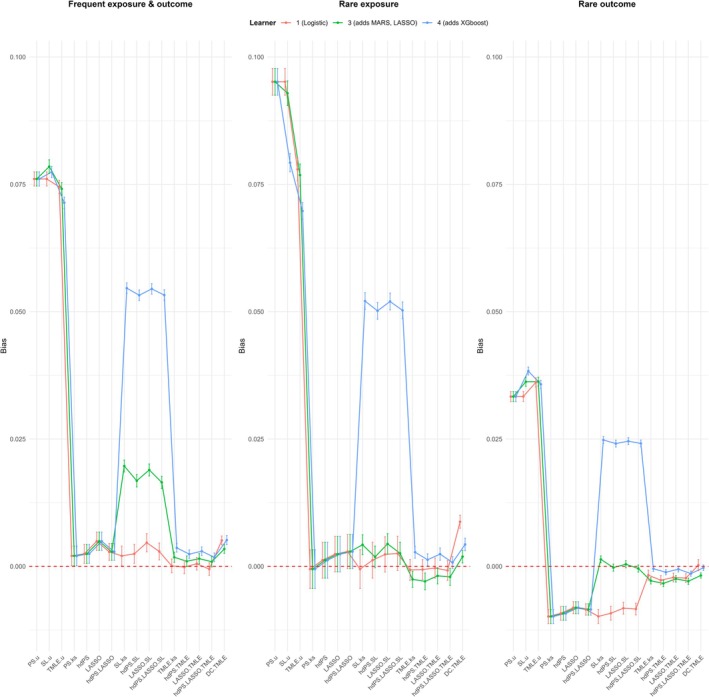
Bias estimates for different methods under frequent, rare exposure, and rare outcome scenarios.

**FIGURE 3:**
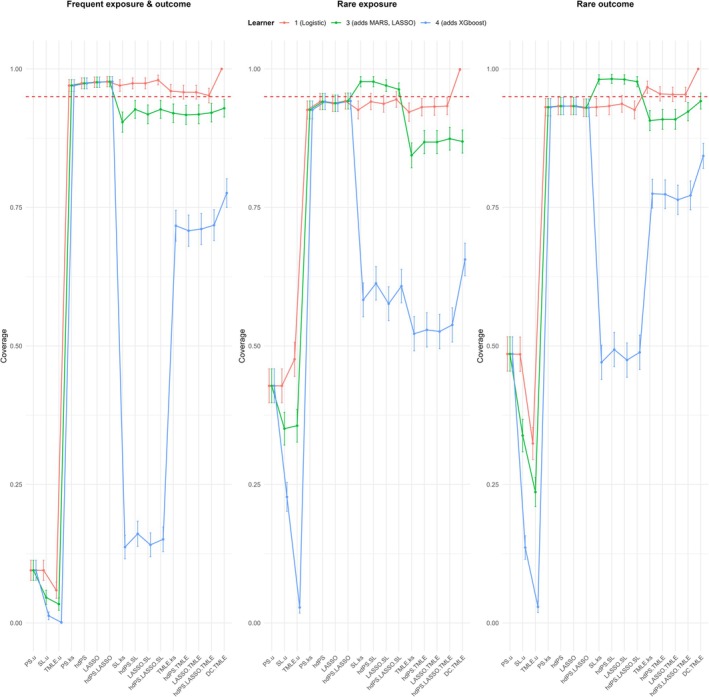
95% coverage estimates for different methods under frequent, rare exposure, and rare outcome scenarios.

Figures 2 and 3 illustrate the bias and 95% coverage estimates across frequent, rare exposure, and rare outcome scenarios. The results are summarized below.

#### 3.2.1 Frequent Exposure and Outcome Scenario

##### 3.2.1.1 Bias

In the frequent exposure and outcome scenario, the bias results demonstrated that the TMLE methods consistently achieved the smallest bias across all 3 libraries considered. With the 3‐learner library, TMLE methods exhibited bias values ranging from 0.001 to 0.002, which were marginally lower than the bias observed for standard methods with proxies (0.002–0.005) and the DC TMLE method (0.003). The SL methods showed a higher bias ranging from 0.016 to 0.02, while methods without proxies had the highest bias (0.074–0.079), as anticipated.

When transitioning to a 4‐learner library, bias for the TMLE methods slightly increased to 0.002–0.004, and the DC TMLE showed a similar trend with a bias of 0.005. Notably, the SL methods demonstrated a significant increase in bias, reaching values of 0.053–0.055. By contrast, when employing a 1‐learner library, the TMLE methods achieved their lowest bias values (−0.0005 to 0.0001), while the bias for SL methods decreased substantially to 0.002–0.005. Adjusting for all measured confounders reduced the bias for SL methods (from 0.009–0.013) by approximately 0.007 and also slightly decreased the bias of standard methods with proxies. Further adjustments for proxies reduced SL bias even more substantially, bringing it to −0.002–0.001.

##### 3.2.1.2 Coverage

The coverage results revealed that methods without proxies achieved the lowest coverage with the 3‐learner library, with values around 6%, indicating highly unreliable estimates. Standard methods with proxies achieved the highest coverage at approximately 97.4%, reflecting well‐calibrated confidence intervals. Other methods, including TMLE and SL approaches, exhibited coverage slightly below the nominal 95% (around 92%), indicating marginally under‐calibrated intervals.

The 4‐learner library produced a dramatic reduction in coverage across all methods. For TMLE and DC TMLE, coverage dropped to 71.4% and 77.6%, respectively, while SL methods experienced a drastic decline to 14.8%. By contrast, with the 1‐learner library, DC TMLE achieved the highest coverage (100%), followed by TMLE (95.7%) and SL methods (97.5%), all exceeding nominal coverage. Adjusting for all measured confounders restored coverage to nominal levels (~95%) for standard methods with proxies but reduced coverage to 89.9% for SL methods.

##### 3.2.1.3 SEs

Empirical SE values indicated that DC TMLE had the lowest variability (0.014) among all methods using the 3‐learner library, followed by TMLE methods (0.016–0.017). SL methods exhibited slightly higher SE values (0.019–0.02), while methods without proxies (0.02–0.022) displayed moderate variability. Standard methods with proxies had the highest empirical SE (0.028–0.031), suggesting greater variability. Switching to the 4‐learner library slightly reduced empirical SE across methods (excluding DC TMLE, which remained unchanged). Conversely, the 1‐learner library increased empirical SE for SL methods by approximately 0.01, while DC TMLE remained consistent. Adjusting for confounders significantly reduced empirical SE for standard methods with proxies.

The relative error in SE results showed that methods involving TMLE approaches had negative values with the 3‐learner library, whereas other methods exhibited positive values. SL methods had the largest magnitude of relative error, with values around 17.5, indicating the poorest performance. In contrast, TMLE methods demonstrated relative errors with smaller magnitudes (~8.2). For the 4‐learner library, relative error for TMLE methods decreased significantly by approximately 34, while for the 1‐learner library, the relative error for DC TMLE increased dramatically to 98.6. Adjustments for measured confounders and proxies shifted the relative error for SL methods and standard methods with proxies to negative values, substantially improving the results.

#### 3.2.2 Rare Exposure and Frequent Outcome Scenario

##### 3.2.2.1 Bias

In this scenario, methods without proxies showed the highest bias (0.077–0.095) using the 3‐learner library, while all other methods had similar, low bias values ranging from −0.003 to 0.004. Switching to a 4‐learner library resulted in a significant increase in bias for SL methods (0.05–0.052), while other methods showed no major changes. Adjusting for all measured confounders led to a slight improvement in bias for standard methods with proxies, reducing it to values between −0.006 and −0.0003.

##### 3.2.2.2 Coverage

Coverage results revealed that only SL methods exceeded nominal coverage with the 3‐learner library, achieving 97.2%, while standard methods with proxies demonstrated slightly under‐calibrated intervals at 93.7%. TMLE and DC TMLE both had similar coverage (~86.3% and ~86.9%, respectively), and methods without proxies had the lowest coverage at 37.8%. Switching to the 4‐learner library resulted in a universal decrease in coverage, with reductions of approximately 30% for learner‐based methods. Adjustments for confounders and proxies improved coverage to nominal levels (~95%) for methods such as PS.u and SL.u.

#### 3.2.3 Frequent Exposure and Rare Outcome Scenario

##### 3.2.3.1 Bias

For this scenario, SL methods demonstrated the lowest bias (−0.0004 to 0.001) using the 3‐learner library, while TMLE methods and DC TMLE had slightly higher negative bias values (−0.003 to −0.002 and −0.002, respectively). Methods without proxies had the largest bias (0.033–0.036). Adjusting for confounders slightly reduced SL bias but did not significantly affect other methods.

##### 3.2.3.2 Coverage

Coverage trends were consistent with prior scenarios. SL methods achieved the highest coverage (~98%) with the 3‐learner library, while TMLE (~91.2%) and DC TMLE (~94.2%) exhibited slightly under‐calibrated intervals. Methods without proxies again had the lowest coverage (~35.3%). Switching to the 4‐learner library resulted in substantial coverage reductions for SL methods, while the 1‐learner library restored coverage for DC TMLE and TMLE to exceed nominal levels.

Adjusting for all measured confounders in the outcome models of non‐TMLE methods with proxies modestly improved bias and coverage for standard methods, while additional proxy adjustments in the outcome model significantly reduced residual confounding, particularly for SL methods. Our results also indicated that stabilizing the weights did not introduce noticeable differences in the trends for bias, coverage, or standard errors across the evaluated metrics in this particular high‐dimensional setting.

Supporting Information: Appendix Figures C.1 and C.2 show the bias and coverage estimates for all scenarios, stratified by the number of learners used in the super learner libraries. More granular results are available in the following web app: ehsank.shinyapps.io/hdPS‐TMLE/.

## 4 Discussion

### 4.1 Motivating Example

Methods that incorporated high‐dimensional proxies, such as standard hdPS, TMLE, and SL methods, yielded similar point estimates for the association between obesity (exposure) and diabetes (outcome), regardless of the candidate learner library used. Methods without proxies showed notable deviations in their estimates. While this motivating example highlighted general trends in estimator performance, it was limited to a single dataset and lacked the capacity to systematically assess bias, variance, and coverage. In contrast, the plasmode simulation provided a comprehensive evaluation of these statistical properties across a range of realistic scenarios, offering deeper insights into estimator behavior in high‐dimensional contexts.

### 4.2 Simulation Results

Our plasmode simulation revealed distinct behaviors across general groups of methods. Methods without proxies (PS.u, SL.u, TMLE.u) performed the worst across all scenarios, showing consistently high bias and severely under‐calibrated coverage. These findings emphasize the critical role of proxies in confounding adjustment, especially in the presence of unmeasured confounding. In contrast, standard methods incorporating high‐dimensional proxies (e.g., PS.ks, hdPS, LASSO, hdPS.LASSO) demonstrated substantial improvements in bias and coverage, achieving near‐nominal coverage in most scenarios.

SL methods (hdPS.SL, LASSO.SL, hdPS.LASSO.SL, SL.ks) showed flexibility and reasonable bias performance but experienced significant coverage deterioration when non‐Donsker learners were included in the library. TMLE methods (hdPS.TMLE, LASSO.TMLE, hdPS.LASSO.TMLE, TMLE.ks) exhibited robust performance with low bias and reliable coverage for smooth learners. However, as with SL methods, coverage markedly declined when complex learners, such as XGBoost, which do not belong to the Donsker class, were included. Among TMLE methods, DC TMLE consistently achieved the lowest empirical SE and well‐calibrated coverage in most scenarios, yet underperformed in high‐dimensional settings when non‐Donsker learners were included.

The SL framework, particularly its integration within the TMLE framework, is widely recognized for its flexibility and ability to incorporate diverse learners [5]. However, our findings highlight key nuances in its application to high‐dimensional proxy adjustment. While previous SL tutorials emphasize the benefits of library diversity [29, 30], our results suggest that increased complexity is not always beneficial. Specifically, simpler libraries, such as the 1‐learner and 3‐learner configurations, consistently outperformed larger libraries in terms of bias and coverage, achieving greater stability across all scenarios. The 1‐learner library avoided overfitting and adhered to theoretical assumptions, while the 3‐learner library (logistic regression, LASSO, and MARS) performed similarly but occasionally exhibited slightly higher bias and under‐calibrated coverage.

Conversely, the 4‐learner library, which included XGBoost (a non‐Donsker learner), introduced significant variability, resulting in increased bias and poor coverage, particularly in rare exposure scenarios. These findings align with guidelines advocating for tailoring SL libraries to effective sample size [15] and underscore the importance of including simpler algorithms, such as regression splines and generalized linear models, in SL libraries [13]. While DC TMLE has demonstrated advantages in low‐dimensional settings [17], its performance in high‐dimensional settings with complex learners was unsatisfactory. This suggests that larger libraries, though flexible, may introduce instability, especially when incorporating learners that violate Donsker conditions.

### 4.3 Model Complexity Versus High‐Dimensionality

The strong performance of standard methods incorporating high‐dimensional proxies (e.g., PS.ks, hdPS, LASSO, hdPS.LASSO) across all scenarios highlights the value of simple yet effective modeling strategies in high‐dimensional settings. These methods benefit from directly leveraging proxies for confounding adjustment, which—when selected through structured algorithms such as hdPS or LASSO—can capture key confounding signals without adding unnecessary complexity. This simplicity mitigates overfitting and instability [31], leading to bias reduction and near‐nominal confidence interval coverage.

While ensemble learners such as SL offer modeling flexibility, they may also introduce greater variance—particularly when combined with unstable or non‐smooth base learners. Similarly, the iterative targeting step of TMLE, though designed to improve efficiency, may exacerbate small‐sample variability in the presence of high‐dimensional covariate spaces or complex learners [32]. For TMLE methods, thoughtful selection of library components (favoring smooth learners) may help mitigate these issues [13, 15].

These observations align with semiparametric theory, which warns that greater model flexibility often comes at the cost of slower convergence rates. Nonparametric and machine learning estimators typically converge more slowly than parametric models, and this can result in inflated estimation error and poor finite‐sample performance—particularly when both treatment and outcome models must be estimated non‐parametrically [33]. This tradeoff is especially critical in doubly robust estimators such as TMLE, which require adequate convergence of both nuisance functions.

These challenges are exacerbated in high‐dimensional settings due to the curse of dimensionality and increased estimation instability. As our results demonstrate, higher modeling complexity does not necessarily lead to improved statistical properties. In fact, it can degrade performance when flexible learners are combined with iterative procedures such as the TMLE targeting step. These findings reinforce theoretical guidance: added flexibility must be carefully balanced against potential variance inflation and instability—especially in complex, high‐dimensional causal inference problems.

### 4.4 Strengths, Limitations, and Future Directions

This study provides a systematic evaluation of high‐dimensional proxy adjustment methods combined with machine learning and doubly robust estimators. By leveraging realistic plasmode simulations, we examined estimator performance across varying exposure and outcome prevalence scenarios. Unlike prior studies that focused on low dimensions or investigator‐specified covariates, our work extends the literature by assessing diverse adjustment strategies and learner configurations. Importantly, we highlight the challenges posed by non‐Donsker learners within the hdPS framework and their impact on bias, coverage, and stability.

However, this study has several limitations. We did not assess overlap between treatment groups or conduct diagnostics, such as covariate balance checks, across all simulations, as recommended by transparency guidelines [34, 35, 36]. Additionally, the findings are shaped by the data structure of the plasmode simulation, which may not fully capture complexities such as time‐varying confounding or extreme sparsity. While our study observed under‐coverage with DC TMLE in high‐dimensional settings, prior work suggests that single cross‐fit TMLE, cross‐validated TMLE (CVTMLE) and its variants (e.g., CVTMLE[Q]) could mitigate these issues by stabilizing variance and improving confidence interval coverage [37].

Some previous studies have included collaborative targeted maximum likelihood estimation (C‐TMLE) [38] as a comparable method to TMLE, with a specific emphasis on its iterative variable selection procedure aimed at reducing confounding bias [10, 39]. We chose not to include this method in our current work due to practical and methodological considerations specific to high‐dimensional proxy adjustment settings.

While C‐TMLE offers theoretical advantages in iterative covariate selection and bias reduction, its computational complexity poses significant challenges in high‐dimensional datasets, where the numbers of covariates and proxies are large. Although scalable versions of C‐TMLE exist, these rely on preordering strategies, such as logistic preordering or partial correlation approaches, which can introduce arbitrary assumptions about the importance or influence of covariates [40]. This ordering could be particularly problematic in high‐dimensional proxy settings, where the relationships among variables can be complex, and any imposed ordering may fail to capture the true confounding structure. We also did not consider deep learning algorithms, such as neural networks [41, 42], due to their complexity and potential instability in high‐dimensional settings.

Screener‐based approaches could also help mitigate instability caused by non‐Donsker learners by reducing dimensionality prior to applying SL [15]. Extending these methods to more complex contexts, such as time‐varying exposures or longitudinal data [43], represents an important direction for future research. Recent work has highlighted unique challenges in these settings, including dynamic confounding and data sparsity [13]. Addressing these complexities will enhance the applicability of these methods to a wider range of epidemiological studies.

## 5 Conclusion

This study underscores the critical role of high‐dimensional proxy adjustment in reducing residual confounding and demonstrates the strong performance of standard methods incorporating proxies, which achieved robust bias and near‐nominal coverage. The simplicity of these methods, combined with structured proxy selection approaches such as hdPS and LASSO, ensures effective adjustment without introducing instability, even in high‐dimensional settings. In contrast, while TMLE methods excelled in bias reduction, their coverage performance was notably worse than standard methods with proxies, particularly when using more complex learner libraries. These findings highlight the importance of tailoring machine learning and doubly robust estimators to specific data contexts, with careful consideration of library composition to mitigate the instability introduced by non‐Donsker learners.

### 5.1 Plain Language Summary

Observational studies often face challenges in estimating causal relationships due to unmeasured confounding—factors that influence both treatment and outcome but are not directly observed. hdPS methods help address this issue by using proxy variables that indirectly capture unmeasured confounders. This study evaluates how well different approaches—ranging from traditional hdPS methods to machine learning‐based estimators—perform under various data conditions. Using realistic simulations, we find that incorporating high‐dimensional proxies significantly improves bias reduction and coverage. While methods such as TMLE and super learner offer advantages, their performance depends on the choice of models. More complex configurations, particularly those including advanced learners such as XGBoost, may introduce instability, leading to reduced accuracy. Our findings suggest that simpler models, including traditional hdPS with proxies, often provide robust and reliable results. We also applied these methods in a real‐world data from the NHANES (2013–2018). These results offer valuable guidance for researchers applying statistical methods to reduce confounding bias in epidemiological studies.

## Author Contributions

**M.E.K.:** conceptualization, writing – original draft, supervision, review and editing. **Y.L.:** formal analysis, review and editing.

## Ethics Statement

The analysis conducted on secondary and de‐identified data is exempt from research ethics approval requirements. Ethics for this study was covered by item 7.10.3 in University of British Columbia's Policy #89: Research and Other Studies Involving Human Subjects 19 and Article 2.2 in the Tri‐Council Policy Statement: Ethical Conduct for Research Involving Humans (TCPS2).

## Consent

The National Health and Nutrition Examination Survey (NHANES), conducted by the U.S. Centers for Disease Control and Prevention (CDC), involves collecting data through direct physical examinations, laboratory testing, and interviews. The CDC already obtains consent from participants when collecting this data. When researchers use NHANES data for their studies, they are typically using de‐identified, publicly available data. This means that the information cannot be linked back to individual participants, and therefore, additional consent from participants is not required for researchers to use this data.

## Conflicts of Interest

M.E.K. is currently supported by grants from Canadian Institutes of Health Research and MS Canada. M.E.K. has previously received consulting fees from Biogen Inc. for consulting unrelated to this current work. M.E.K. was also previously supported by the Michael Smith Foundation for Health Research Scholar award.

## Supporting information


**Data S1.** Supporting Information.

## Data Availability

NHANES data is publicly accessible and can be retrieved from the NHANES website. The datasets generated and/or analyzed during the current study are available in the NHANES repository at www.cdc.gov.
