# Supplementary material for: How Effective Are Machine Learning and Doubly Robust Estimators in Incorporating High‐Dimensional Proxies to Reduce Residual Confounding?
Source: Pharmacoepidemiol Drug Saf. 2025 May 14;34(5):e70155. doi: 10.1002/pds.70155 (PMC12076102; doi:10.1002/pds.70155)
Supplement: Supplementary file 1 — Data S1. Supporting Information. [file PDS-34-e70155-s001.pdf]

Supplementary Content

How Effective Are Machine Learning and Doubly Robust Estimators in Incorporating High-Dimensional Proxies to Reduce Residual Confounding?

A Details of methods under consideration

A.1 High-dimensional propensity score (hdPS) algorithm

The high-dimensional propensity score (`hdPS`) algorithm (Schneeweiss et al., 2009) is a semi-automated approach for confounding adjustment using routinely collected healthcare data. It proceeds in five main steps: (1) define multiple data dimensions (e.g., inpatient diagnoses, outpatient procedures, medications); (2) within each dimension, optionally select the top n most prevalent codes; (3) transform these codes into binary variables indicating their presence or frequency for each individual; (4) rank all candidate covariates using the Bross formula, which estimates the potential for confounding bias; and (5) select the top-ranked variables to include in the propensity score model, along with investigator-specified covariates. This approach enables the inclusion of rich proxy information that may capture unmeasured confounding.

In our implementation, binary proxy variables were first filtered to remove those with no variation (i.e., all 0s or all 1s). We then applied the `get_prioritised_covariates()` function from the `autoCovariateSelection` package, which ranks variables using Bross's bias formula based on their marginal associations with treatment and outcome. The top 100 ranked proxies were retained and combined with investigator-specified covariates (e.g., demographic and laboratory variables) to form the final covariate set. Propensity score weights were then estimated using this set of variables, followed by outcome regression to estimate treatment effects (e.g., risk differences, using `WeightIt` with `method = "ps"`). This implementation preserves the key structure of the original hdPS algorithm while adapting it to support weighting-based estimators.

For all non-Targeted Maximum Likelihood Estimation (TMLE) methods (hdPS, LASSO- and super learning [SL]-based), treatment effects were estimated using outcome regression models following inverse probability weighting. Specifically, multiple versions of weighted linear regression models were fit using the `glm()` function with a Gaussian identity link (Naimi and Whitcomb, 2020). All models were fit using both unstabilized and stabilized weights. Robust standard errors were computed using the `sandwich` package. These models varied based on which covariates were included, and the details are listed in Appendix Table A.1.

A.2 Propensity score models with and without proxies

The `PS.ks` and `PS.u` methods represent standard propensity score (PS) models that differ in their use of proxy variables. Both models estimate the PS using investigator-specified baseline covariates (e.g., demographics and lab variables), but only `PS.ks` incorporates additional high-dimensional proxy variables (i.e., empirical covariates). In our implementation, `PS.ks` uses the full set of proxy variables (`proxy.list`) together with the baseline covariates to estimate the propensity score and generate inverse probability weights. In contrast, `PS.u` omits all proxy variables by setting `proxy.list.sel = NULL` during weight estimation, thereby relying solely on investigator-specified covariates.

A.3 LASSO-based methods

`LASSO` is a penalized regression approach that performs variable selection based on the predictive utility of covariates for the outcome. In our implementation, we applied logistic LASSO using `glmnet::cv.glmnet`, incorporating all available covariates—investigator-specified variables (e.g., demographics and labs) and all empirical proxy

Appendix Table A.1: Outcome model covariate adjustment strategies used across high-dimensional propensity score and alternative analytic methods (super learning- and LASSO-based). Each strategy varies in the inclusion of investigator-specified baseline covariates and proxy variables, and whether selection was based on post-weighting covariate imbalance.

Adjustment Strategy	Investigator-Specified Covariates	Proxies	Notes
No Adjustment	×	×	Only the exposure variable was included in the outcome model.
All Baseline Covariates (Ho et al., 2007)	✓	×	Included all investigator-specified demographic and laboratory covariates, regardless of balance.
All Covariates and Selected Proxies	✓	✓	Included all baseline covariates plus proxy variables selected by the specific method (e.g., hdPS or LASSO).
Only Imbalanced Covariates (Nguyen et al., 2017)	✓ (SMD $>$ 0.1)	×	Included baseline covariates with post-weighting standardized mean differences (SMDs) $>$ 0.1.
Only Imbalanced Covariates and Proxies	✓ (SMD $>$ 0.1)	✓ (SMD $>$ 0.1)	Included both baseline and selected proxy variables with SMD $>$ 0.1 after weighting.

Abbreviations: SMD, standardized mean difference; hdPS, high-dimensional propensity score; LASSO, least absolute shrinkage and selection operator.

- Targeted Maximum Likelihood Estimation (TMLE)-based methods are not included in this table because they rely on the same covariate set for both the treatment and outcome models, and do not involve separate adjustment strategies for outcome modeling.

variables—as predictors of the outcome (Karim et al., 2018; Franklin et al., 2015). The algorithm selects variables by shrinking the coefficients of weak predictors toward zero, retaining only those with non-zero coefficients at the optimal penalty (`lambda.min`). All selected proxies, along with predefined covariates (investigator-specified variables), were used for propensity score estimation. Unlike hdPS, which ranks covariates based on joint associations with treatment and outcome, LASSO focuses entirely on outcome prediction.

The `hdPS.LASSO` method is a hybrid variable selection strategy that combines the strengths of `hdPS` and `LASSO`. It begins by applying the hdPS algorithm to prioritize empirical proxies using the Bross bias formula, which ranks variables based on their joint associations with treatment and outcome. The top 100 hdPS-ranked proxies are retained as candidate variables. Then, logistic LASSO (`glmnet::cv.glmnet`) is applied to this reduced subset of proxies along with the outcome, selecting a final set of proxies with non-zero coefficients. This two-stage procedure first filters variables likely to be confounders and then applies outcome-based shrinkage to refine the set. The resulting proxies, along with investigator-specified covariates, are used to estimate propensity scores. This hybrid method balances confounding bias reduction (via `hdPS`) and parsimony or model sparsity (via `LASSO`).

A.4 SL-based methods

All SL-based methods in this study (`hdPS.SL`, `LASSO.SL`, `hdPS.LASSO.SL`, and `SL.ks`) share a common structure: they use SL to estimate the propensity score based on selected covariates, and then apply inverse probability weighting to estimate treatment effects (Karim, 2025). These methods differ only in how proxy variables are selected prior to SL modeling. Specifically, `hdPS.SL` uses proxies selected via the `hdPS` algorithm (Bross ranking using the `get_prioritised_covariates()` function and the top 100 ranked proxies are selected), `LASSO.SL` applies penalized logistic regression (`LASSO`) for selection, `hdPS.LASSO.SL` combines both (`hdPS` ranking followed by `LASSO` refinement) in selecting the proxies, and `SL.ks` includes all proxies without selection. After variable selection, SL is used as a flexible, ensemble-based alternative to logistic regression for modeling treatment assignment, potentially

capturing nonlinearities and interactions (Phillips et al., 2023). Thus, the key distinction lies not in variable selection but in the modeling algorithm used for treatment assignment: parametric vs. ensemble learning.

A.5 TMLE-based methods

All TMLE-based methods in this study (`hdPS.TMLE`, `LASSO.TMLE`, `hdPS.LASSO.TMLE`, and `TMLE.ks`) share a common framework for causal estimation: they use TMLE to combine flexible machine learning–based outcome modeling with propensity score estimation (Karim, 2025). The key difference across these methods lies in the variable selection step. Each method selects empirical proxy variables using a different strategy—Bross ranking (`hdPS.TMLE`), outcome-driven LASSO (`LASSO.TMLE`), a hybrid of the two (`hdPS.LASSO.TMLE`), or no selection (`TMLE.ks`). After selection, all methods estimate the treatment model (propensity score) using Super Learner via `WeightIt` and pass both the selected covariates and the estimated g-function to the `tmle()` function. This ensures doubly robust estimation, allowing both models (treatment and outcome) to be flexibly specified. Thus, while these TMLE variants differ in the covariates used, they follow an identical structure in how TMLE is executed, leveraging Super Learner.

The `DC.TMLE` method implements a cross-validated, repeated sample-splitting version of TMLE, designed to reduce bias and improve finite-sample performance (Mondol and Karim, 2024). It performs double cross-fitting by splitting the data into $K = 3$ mutually exclusive folds (`n_split = 3`) (Zivich and Breskin, 2021) and repeating the procedure 25 times (`num_cf = 25`) (Karim and Mondol, 2025), each with a different random seed to ensure variability in the splits. In each iteration, the treatment (propensity score) and outcome models are estimated separately using Super Learner with a fixed library of machine learning algorithms. The clever covariates are computed using predicted probabilities from held-out folds, and fluctuation steps are performed using logistic regression to update the initial outcome predictions. Each split yields a TMLE estimate of the average treatment effect (ATE) and its influence function-based variance. The final effect estimate is calculated by aggregating the ATEs across all repetitions—using the median—and combining the corresponding variances to obtain a robust standard error that reflects both estimation and resampling variability. This approach helps mitigate overfitting and instability common in high-dimensional estimation and is particularly effective when paired with flexible learners. In contrast, standard TMLE performs a single round of estimation without repeated splitting.

B Details of Plasmode simulation

B.1 Variables Used for Plasmode Simulation Data Generation

The NAHNES dataset is used as an inspiration for the plasmode simulation. It includes a comprehensive set of variables spanning demographic, behavioral, health history, laboratory, and prescription code domains.

Demographic variables (8 total) capture key sociodemographic characteristics, such as age, sex, education level, race/ethnicity, marital status, income, country of birth, and survey cycle. **Behavioral variables** (5 total) reflect lifestyle and health-related habits, including smoking status, dietary habits, high cholesterol status, physical activity level, and sleep patterns. Additionally, **health history and access variables** (2 total) account for background health conditions and healthcare access, specifically family history of diabetes and access to medical care.

In a real-world data analysis context, it is possible that analyst may not know the true model-specification. To mimic that, six **transformed laboratory variables** were derived from original lab measures, such as uric acid, protein, bilirubin, phosphorus, sodium, potassium, globulin, calcium, systolic blood pressure, and diastolic blood pressure. These transformations include logarithmic, multiplicative, and ratio-based operations. Exact transformation formats are as follows: `log(globulin)`, `protein × calcium`, `(diastolic blood pressure / systolic blood pressure)²`, `sqrt(uric acid + bilirubin) / 2`, `phosphorus² / (sodium × potassium)`, and `log(systolic blood pressure + 10)`. These transformations aim to capture complex, non-linear relationships between laboratory measures and the outcome. In the process of data analysis, analyst only has access to original lab measures, not the transformed variables.

Finally, in a high-dimensional setting, it is common to have a large number of proxy variables, many of which may be purely noise variables. However, analyst usually does not know which of these are relevant. In our case, comorbidity burden is a key confounder that is not directly observed in the data. To account for this,

a **count-based prescription code variable** was created by summing selected ICD-10-CM codes (converted to recurrence covariates) with values outside the range of 0.8 to 1.2 relative to the outcome. This variable, represented as $\sum_{s=1}^{94} R_s$, serves as a proxy for comorbidity burden.

B.2 True Outcome Model for Plasmode Simulation Data Generation

The diabetes outcome in the plasmode simulation is modeled as a function of obesity (the exposure), along with demographic, behavioral, and health history variables, transformed laboratory variables, and the count-based prescription code variable. This model aims to reflect the complex interplay of sociodemographic, behavioral, clinical, and laboratory factors in predicting diabetes risk, while also accounting for the influence of comorbidity burden as captured by the prescription code variable.

C Bias and Coverage Estimates Across Methods and Super Learner Configurations

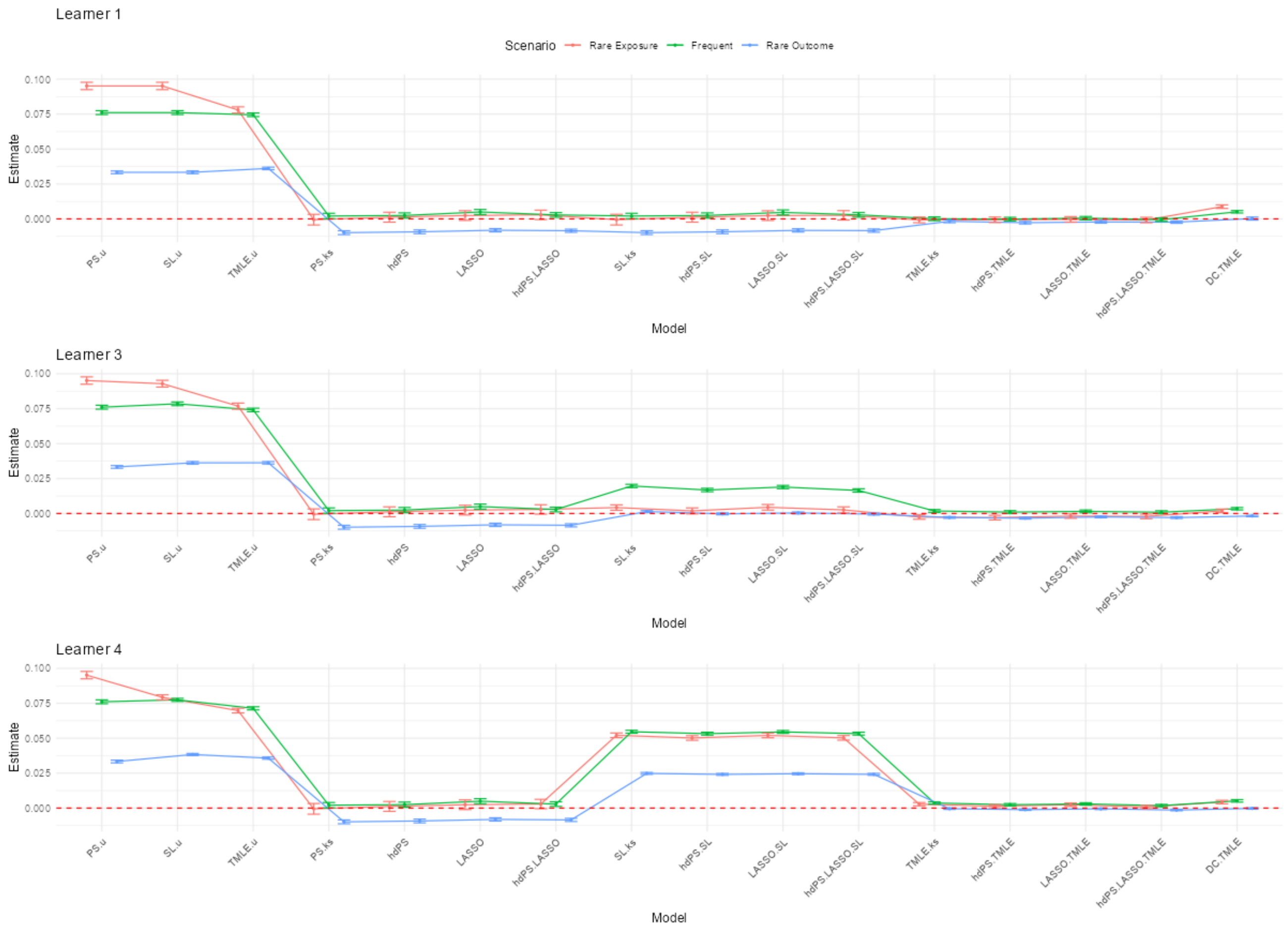

Appendix Figure C.1: Bias estimates for various methods under three super learner configurations: 1 learner (logistic regression), 3 learners (logistic regression, MARS, and LASSO), and 4 learners (adding XGBoost, a non-Donsker learner). The same super learners were applied in TMLE methods. Standard methods, which did not rely on super learners, demonstrated robust performance with high-dimensional proxies (e.g., hdPS, LASSO).

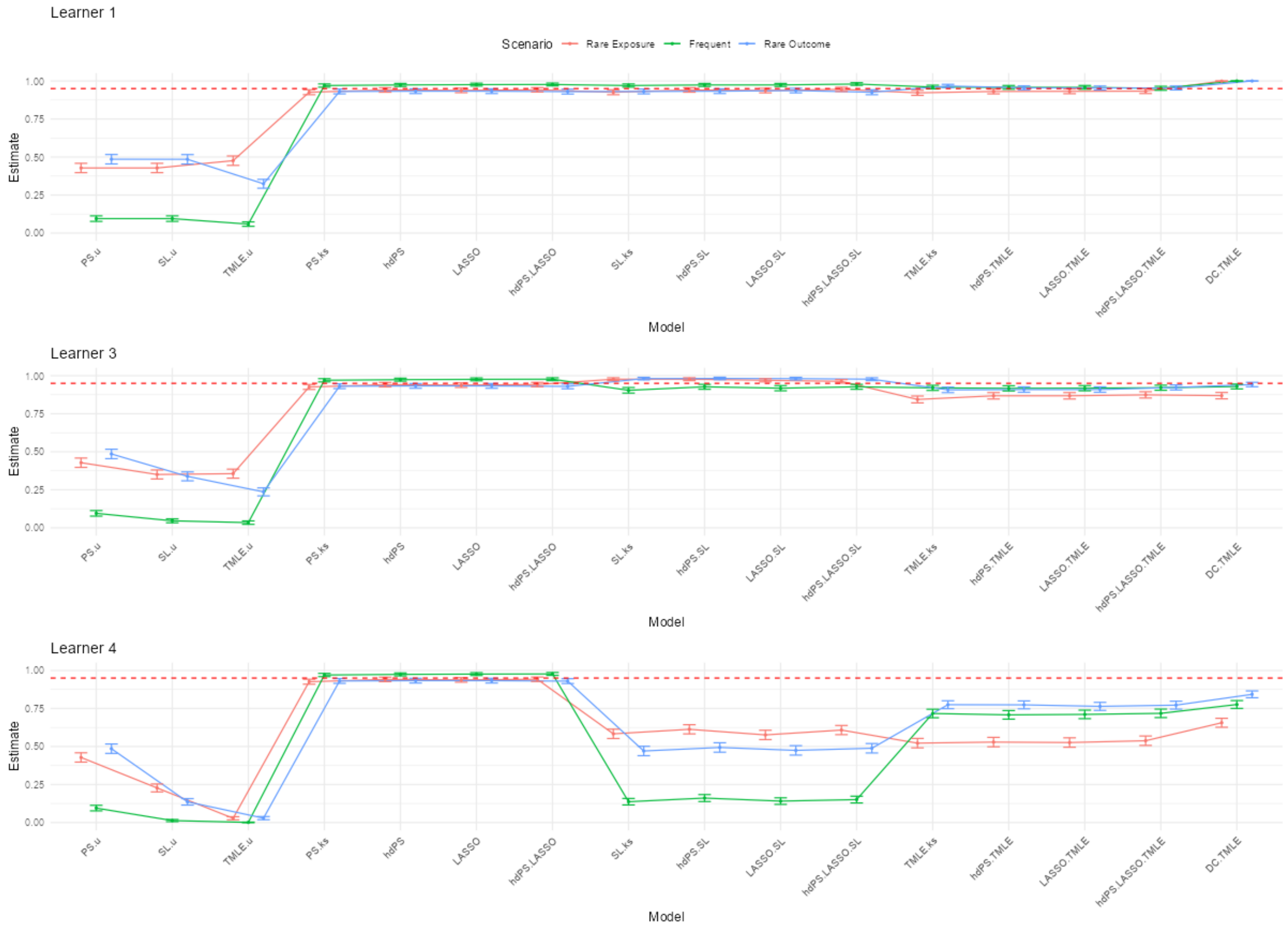

Appendix Figure C.2: Coverage estimates (nominal level: 95%) for various methods under three super learner configurations: 1 learner (logistic regression), 3 learners (logistic regression, MARS, and LASSO), and 4 learners (adding XGBoost, a non-Donsker learner). The same super learners were applied in TMLE methods. Standard methods, which did not rely on super learners, demonstrated robust performance with high-dimensional proxies (e.g., hdPS, LASSO).

Reference

Jessica M Franklin, Wesley Eddings, Robert J Glynn, and Sebastian Schneeweiss. Regularized regression versus the high-dimensional propensity score for confounding adjustment in secondary database analyses. *American journal of epidemiology*, 182(7):651–659, 2015.

Daniel E Ho, Kosuke Imai, Gary King, and Elizabeth A Stuart. Matching as nonparametric preprocessing for reducing model dependence in parametric causal inference. *Political analysis*, 15(3):199–236, 2007.

Mohammad Ehsanul Karim. High-dimensional propensity score and its machine learning extensions in residual confounding control. *The American Statistician*, 79(1):72–90, 2025.

Mohammad Ehsanul Karim and Momenul Haque Mondol. Finding the optimal number of splits and repetitions in double cross-fitting targeted maximum likelihood estimators, 2025. Manuscript under review.

Mohammad Ehsanul Karim, Menglan Pang, and Robert W Platt. Can we train machine learning methods to outperform the high-dimensional propensity score algorithm? *Epidemiology*, 29(2):191–198, 2018.

Momenul Haque Mondol and Mohammad Ehsanul Karim. Towards robust causal inference in epidemiological research: Employing double cross-fit tmle in right heart catheterization data. *American Journal of Epidemiology*, page kwae447, 2024.

Ashley I Naimi and Brian W Whitcomb. Estimating risk ratios and risk differences using regression. *American journal of epidemiology*, 189(6):508–510, 2020.

Tri-Long Nguyen, Gary S Collins, Jessica Spence, Jean-Pierre Daurès, PJ Devereaux, Paul Landais, and Yannick Le Manach. Double-adjustment in propensity score matching analysis: choosing a threshold for considering residual imbalance. *BMC medical research methodology*, 17:1–8, 2017.

Rachael V Phillips, Mark J van der Laan, Hana Lee, and Susan Gruber. Practical considerations for specifying a super learner. *International Journal of Epidemiology*, 2023. doi: 10.1093/ije/dyad023.

Sebastian Schneeweiss, Jeremy A Rassen, Robert J Glynn, Jerry Avorn, Helen Mogun, and M Alan Brookhart. High-dimensional propensity score adjustment in studies of treatment effects using health care claims data. *Epidemiology*, 20(4):512, 2009.

Paul N Zivich and Alexander Breskin. Machine learning for causal inference: on the use of cross-fit estimators. *Epidemiology*, 32(3):393, 2021.
